# The complete mitochondrial transcript of the red tail loach *Yasuhikotakia modesta* as assembled from RNAseq (Teleostei: Botiidae)

**DOI:** 10.1080/23802359.2016.1275851

**Published:** 2017-02-02

**Authors:** José Horacio Grau, Leon Hilgers, Janine Altmüller, Vendula Šlechtová, Jörg Bohlen

**Affiliations:** a Museum für Naturkunde Berlin, Leibniz-Institut für Evolutions- und Biodiversitätsforschung, Berlin, Germany;; b Cologne Center for Genomics (CCG), Universität zu Köln, Köln, Germany;; c Laboratory of Fish Genetics, Institute of Animal Physiology and Genetics, Czech Academy of Sciences, Liběchov, Czech Republic

**Keywords:** *Yasuhikotakia modesta*, loach, Botiidae, mitogenome

## Abstract

Here, we present the first complete mitochondrial genome of the Red Tailed Loach *Yasuhikotakia modesta* (Teleostei: Botiidae) from Thailand, assembled from next-generation transcriptome sequencing data. The assembled transcript corresponds to the full length mitochondrial genome of *Y. modesta*, which measured 16,865 bp in length, and contained 13 protein-coding genes, 2 ribosomal RNA genes, and 22 transfer RNA genes. A slight A + T bias was observed in the mitogenome of *Y. modesta* with an overall base composition of 32.2% A, 25.8% T, 26.4% C, and 15.4% G, and a GC content of 41.8%. The gene arrangement was identical to that of previously described loach mitogenomes.

Freshwater fishes of the family Botiidae contain eight genera and 80 species which are widespread across South-, Southeast-, and East Asia. They are high-priced for their taste and a number of species, especially from the genera *Botia, Chromobotia,* and *Yasuhikotakia* are famous in ornamental fish keeping. One of the two subfamilies of Botiidae, the Botiinae, contains only tetraploid species, but details concerning this polyploidization remain unknown (Šlechtová et al. 2006). The reconstruction of a reliable phylogeny of Botiidae will be best achieved with the use of genetic data, including mitochondrial genes. Here, we present the full mitogenome of *Y. modesta*, a tetraploid species that occurs in the Mekong River and Chao Phraya River (Nalbant 2002).

The specimen was collected near the Mekong mainstream at the locality of Ubon Ratchathani (15 20′16″N 105 28′00″E) and deposited in the collection of the Institute of Animal Physiology and Genetics in Liběchov, Czech Republic, under the accession number A9732.

A full-length transcript of the mitogenome was obtained from the next-generation transcriptome sequencing. Paired end Illumina sequencing libraries were generated from 600ng total RNA input with fragment sizes of 260 bp as described by Gao et al. (2014). The libraries were sequenced on an Illumina HiSeq2000 platform using Illumina RNA TruSeq protocol and kit v2. Sequencing yielded 117,040,239 100 bp paired end reads, which were used for a *de novo* transcriptome assembly of *Y. modesta* with SOAPtransdenovo2 (v240) (Xie et al. 2014) and parameters -K 31 -M 3 -F. The assembled mitogenome transcript was manually inspected for repeats at the transcript ends to confirm circularity. Annotations were carried out with MITOchondrial genome annotation Server (MITOS) (Bernt et al. 2012), and manual validation of the coding regions using the NCBI ORF Finder (http://www.ncbi.nlm.nih.gov/gorf/gorf.html).

The annotated sequence file was submitted to NCBI (accession no. KY131962). The phylogenetic position of the new sequence of *Y. modesta* according to the gene Cytochrome B is shown in Figure 1.

**Figure 1.**
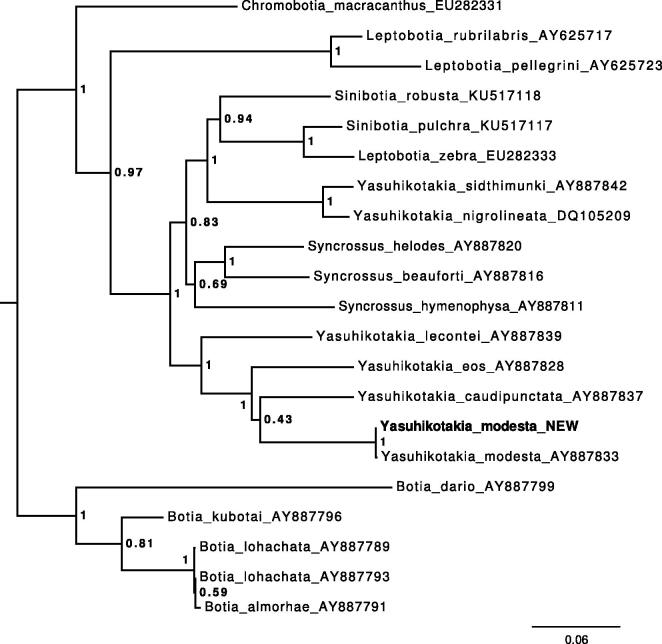
Maximum-likelihood tree illustrating the phylogenetic position of the newly sequenced *Y. modesta* gene sequence among a subset of Botiidae species. Cytochrome B sequences were aligned using MAFFT 7.271 and highly divergent or poorly aligned regions were removed with Gblocks 0.91b (Castresana 2000) allowing for gap positions and smaller blocks. Trees were calculated using PhyML 3.1 (Guindon et al. 2010) with 12 rate categories, optimized equilibrium frequencies, GTR model of sequence evolution and combined heuristics (nearest neighbour interchange and subtree pruning and rerafting).

The complete mitochondrial transcript of *Y. modesta* was 16,865 bp in length and contained 13 protein-coding genes (PCGs), 2 ribosomal RNA genes, and 22 transfer RNA genes. As described for other fish mitogenomes (Yu et al. 2016), the mitochondrial genome of *Y. modesta* contained a slight A + T bias with an overall base composition of 32.2% A, 25.8% T, 26.4% C, and 15.4% G. The gene arrangement of the present mitogenome is similar to those of other loaches (Wang et al. 2012; Tang et al. 2013). Most of the genes were encoded on the L-strand with the exception of *ND6* and eight tRNA genes (*tRNA^Gln^, tRNA^Ala^, tRNA^Asn^, tRNA^Cys^, tRNA^Tyr^, tRNA^Ser2^, tRNA^Glu^*, and *tRNA^Pro^*) which are encoded in the H-strand. All PCGs had ATG as initiation codon with the exception of *CYTB*, which used GTG as initiation codon. TAA was the most used termination codon with the exception of *COX3*, *CYTB*, *ND2*, *ATP8*, and *ND3*, which used a TAG termination codon. The *12S* and *16S* genes had a length of 947 and 1673 bp, respectively.
